# Fipronil prevents transmission of Lyme disease spirochetes

**DOI:** 10.1017/S0031182024001136

**Published:** 2024-08

**Authors:** Radek Šíma, Adéla Palusová, Tereza Hatalová, Luise Robbertse, Petra Berková, Martin Moos, Petr Kopáček, Veronika Urbanová, Jan Perner

**Affiliations:** 1 Institute of Parasitology, Biology Centre of the Czech Academy of Sciences, České Budějovice, Czech Republic; 2 Biopticka Laborator, Plzen, Czech Republic; 3 Institute of Entomology, Biology Centre of the Czech Academy of Sciences, České Budějovice, Czech Republic

**Keywords:** acaricide, *Borrelia afzelii*, *ex vivo* membrane blood feeding, fipronil, ivermectin, *Ixodes ricinus*, Lyme disease, spirochetes, ticks

## Abstract

Lyme disease, a tick-borne illness caused by *Borrelia* spirochetes, poses a significant threat to public health. While acaricides effectively control ticks on pets and livestock, their impact on pathogen transmission is often unclear. This study investigated the acaricidal efficacy of fipronil against *Ixodes ricinus* ticks and its potential to block *Borrelia afzelii* transmission. Initially, we employed the *ex vivo* membrane blood-feeding system to assess the dose–response acaricidal activity of ivermectin, fipronil and its metabolite fipronil sulfone, when supplemented in the blood meal throughout tick feeding. To obtain the temporal resolution of their acaricidal activity, ticks were allowed to initiate blood feeding on an artificial membrane before being exposed to a 1-time topical application of these acaricides. Fipronil demonstrated superior speed of acaricidal activity, with onset of tick moribundity within a few hours, prompting its selection for further *in vivo* testing with *Borrelia*-infected ticks. The *I. ricinus* nymphs infected with *B. afzelii* were topically treated with fipronil shortly after attachment to mice. Four weeks post-feeding, the skin and internal organs were examined for the presence of *Borrelia*. No spirochetes were detected in any organ of mice exposed to fipronil-treated ticks, while 9 out of 10 control mice, exposed to non-treated infectious ticks, displayed *Borrelia* infection. The *in vitro* co-culture experiments confirmed that fipronil had no direct effect on *Borrelia* viability, indicating a tick-directed effect. Overall, these results underline the potential of fipronil as a valuable tool for tick control strategies and suggest a concept for acaricide-mediated *Borrelia*-transmission blockers.

## Graphical abstract

**Figure:**
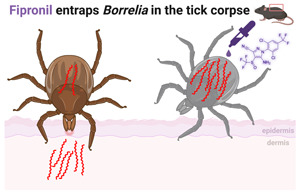

## Introduction

Lyme disease (borreliosis) is the most prevalent vector-borne disease in both Europe and the United States, presenting a significant public health concern. The main causative agent in the United States is *Borrelia burgdorferi*, while the predominant species in Europe are *Borrelia afzelii*, *Borrelia garinii,* and *B. burgdorferi* (Marques *et al*., 2021). Despite its widespread impact, there exist no preventive measures to control Lyme disease. The only FDA-approved Lyme vaccine, LYMErix, targeting outer surface protein A (OspA), was withdrawn from the market in 2002 due to low demand for the vaccine (Nigrovic and Thompson, 2006). However, the increasing number of cases over the last 20 years and growing public awareness have encouraged researchers that there is a market for Lyme prophylactics. A second generation of OspA-based vaccines is currently in development, with the most advanced candidate, VLA15, entering phase 3 trials (Kamp *et al*., 2020; Bézay *et al*., 2023).

In contrast, chemical acaricides have long been proven effective in reducing tick infestation loads on livestock and pets (Obaid *et al*., 2022), primarily by targeting the tick central nervous system (Waldman *et al*., 2023). Fipronil and ivermectin are widely used due to their strong effect against a broad spectrum of pests and parasites. Fipronil belongs to the chemical class of phenylpyrazoles, and its mode of action involves disrupting the arthropod nervous system by blocking gamma-aminobutyric acid chloride channels (Le Corronc *et al*., 2002; Zhao *et al*., 2003). Ivermectin belongs to the class of macrocyclic lactone and targets the glutamate-gated chloride channels, which also leads to paralysis and death of the parasitic organism (Narahashi *et al*., 2010; Campbell, 2012).

In previous studies, passive topical application of fipronil significantly reduced the burden of nymphs and larvae of *Ixodes scapularis* on small reservoir hosts and decreased the abundance of nymphs in treated areas. In addition, infection rates of *B. burgdorferi* and *Anaplasma phagocytophilum* in reservoir animals were significantly reduced after treatment (Dolan *et al*., 2004, 2016). Recent research has also focused on the systemic control of ticks using oral acaricide baits. Studies have demonstrated that oral fipronil bait effectively controls larval *I. scapularis* ticks on white-footed mice (*Peromyscus leucopus*). The efficacy of fipronil has been observed in both laboratory and simulated field conditions, resulting in significant reductions in tick infestations (Poché *et al*., 2020, 2021, 2023*a*). These data indicate that applying acaricides to reservoir animals can represent means to control ticks and interrupt the transmission cycles of tick-borne pathogens. What has remained largely unknown though is whether the currently available acaricides act fast enough to prevent the transmission of tick-borne pathogens. As ticks feed on their host for days, there are inherent waves of pathogen transmission dynamics throughout the course of tick feeding (Eisen, 2018; Pospisilova *et al*., 2019). In this study, we investigated the acaricidal effects of fipronil and ivermectin on feeding *I. ricinus* ticks and tested the ability of fipronil-exposed *I. ricinus* nymphs to transmit *B. afzelii* spirochetes to laboratory mice.

## Materials and methods

### Laboratory animals

*Ixodes ricinus* females were collected by flagging in forests around Ceske Budejovice, Czech Republic. Nymphs and non-infected *I. ricinus* larvae were obtained from the tick rearing facility of the Institute of Parasitology, Biology Centre, Czech Academy of Sciences. All tick life stages were maintained under controlled conditions (temperature: 24°C, humidity: 95%, day/night period: 15/9 h). Inbred, pathogen-free C3H/HeN mice (The Jackson Laboratory, Bar Harbor, ME, USA) were used for the transmission experiments.

All laboratory animals were treated in accordance with the Animal Protection Law of the Czech Republic No. 246/1992 Sb., ethics approval No. 50-2022-P. The study was approved by the Institute of Parasitology, Biology Centre CAS and Central Committee for Animal Welfare, Czech Republic (Protocol No. 1/2015).

### The *ex vivo* membrane feeding

Membrane feeding of ticks was performed using a 6-well plate format according to Kröber and Guerin (Kröber and Guerin, 2007). Whole bovine blood was collected in a local slaughterhouse and manually defibrinated. For feeding of adult *I. ricinus* females, 13 females were placed, together with identical number of males, in a feeding unit. For feeding of *I. ricinus* nymphs, 3 *I. ricinus* females were put in the feeding unit to pre-feed, and on day 1 of feeding, 25 nymphs were put in the feeding unit. All feeding units were lined with a thin silicone membrane of ~80 μm. Blood meals were exchanged every 12 h together with new 6-well plates. All blood meals were supplemented with gentamicin (10 μg mL^−1^; final concentration). For oral exposure *via* blood meal spiking, ivermectin (Merck, Sigma-Aldrich I8898, Germany), fipronil (Merck; Supelco 16785, Germany) and fipronil sulfone (Merck; Supelco 32333, Germany) were solubilized in DMSO as 25 mM stocks. These stocks were then diluted in DMSO in a concentration series and used in for blood meal supplementation (0.1% DMSO, final concentration). Supplementation of the blood meals with acaricides started 48 and 24 h after adding *I. ricinus* adults and nymphs, respectively, into the feeding unit to allow a maximum attachment rate. For topical applications, *I. ricinus* nymphs were allowed to attach to the silicone membrane and feed for 3 days. The acaricidal compounds were solubilized in ethanol as 15 mM solutions (134 μg fipronil in 20 μL and 263 μg ivermectin in 20 μL) and applied on feeding tick nymphs. Nymphs of *I. ricinus* were monitored after topical exposure for viability. The type of movement the ticks exhibited in response to exhaled breath determined their categorization into live, moribund or dead. Ticks were observed 24 h post exposure by Leica Z16 APO macroscope and imaged by Leica DMC6200 Digital Camera. The short videos were recorded by a mobile phone and processed by Adobe Express.

### LC-MS stability assay of fipronil and ivermectin in blood *in vitro*

**Figure 1.**
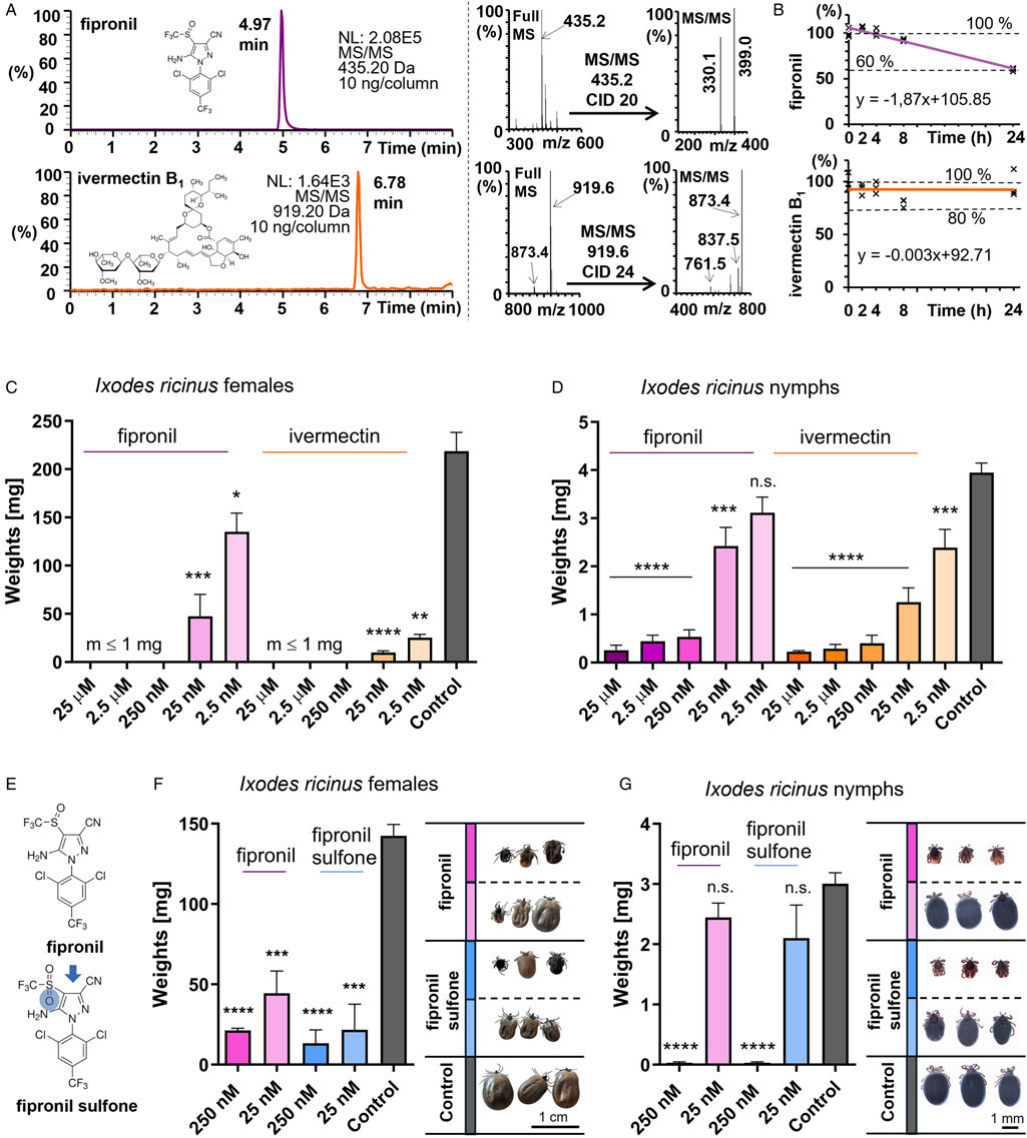
Assessment of stability and acaricidal activity of fipronil and ivermectin in the *ex vivo* blood-feeding system. (A) Chromatogram of fipronil (RT: 4.97 min), and ivermectin B1 (RT: 6.78 min); top right: full MS spectrum of fipronil, the parent ion [M-H]^-^ (435.2 Da) is highlighted in bold; the MS/MS spectrum of 435.2 [M-H]^-^, with highlighted diagnostic/daughter ions (399.0 and 330.1 Da); bottom right: full MS spectrum of ivermectin B1, the parent ions (adducts) [M + Formic-H]^-^ (919.2 Da) and [M-H]^-^ (873.4 Da) are highlighted in bold, the MS/MS spectrum [M + Formic-H]^-^ with highlighted diagnostic/daughter ions (873.4, 837.5, 761.5 Da). (B) Stability curves of fipronil and ivermectin in blood sera *in vitro* incubated at 37°C. (C) Weights of *I. ricinus* females after full engorgement of controls. Final concentrations in the blood meal are shown. Controls were fed blood supplemented with 0.1% DMSO (solvent control); *n* ⩾ 5, mean and standard error of means are shown. *T*-test *P* values: *⩽0.05, **⩽0.01, ***⩽0.001 and ****⩽0.0001. (D) Weights of *I. ricinus* nymphs after full engorgement of controls. Final concentrations in the blood meal are shown. Controls were fed blood supplemented with 0.1% DMSO (solvent control); *n* ⩾ 7, mean and standard error of means are shown. *T*-test *P* values: ***⩽0.001, ****⩽0.0001 and n.s.: *P* = 0.0511. Images from feeding units throughout the feeding of *I. ricinus* nymphs on acaricide-supplemented blood meals are shown as Supplementary Figs S1 and S2. (E) A scheme of the metabolism of fipronil to fipronil sulfone as it occurs in vertebrates. (F) Left: weights of *I. ricinus* adults compared to full engorged controls. Final concentrations in the blood meal are shown. Controls were fed blood supplemented with 0.1% DMSO (solvent control). Ticks were collected after 10 days of feeding. Weights in control group are from spontaneously detached ticks. Mean and standard error of means are shown, *n* ⩾ 5. Right: photographic images of 3 representative *I. ricinus* females at the end of tick *ex vivo* blood feeding with fipronil and fipronil sulfone supplementation. (G) Left: weights of *I. ricinus* nymphs compared to full engorged controls. Final concentrations in the blood meal are shown. Controls were fed blood supplemented with 0.1% DMSO (solvent control). Mean and standard error of means are shown, *n* ⩾ 6. *T*-test *P* values: ****⩽0.0001; n.s. fipronil, *P* = 0.0684; n.s. fipronil sulfone, *P* = 0.0561. Right: photographic images of 3 representative *I. ricinus* nymphs at the end of tick *ex vivo* blood feeding with fipronil and fipronil sulfone supplementation.

Blood sera were obtained by 2 500 × *g* centrifugation of manually defibrinated bovine blood (see above). Sera were filter-sterilized (0.22 μm) and supplemented with 1 mg of fipronil and ivermectin per mL of sera in multiple aliquots for given timepoints. Immediately after taking the samples from given thermal incubation time points (0, 2, 4, 8, 24 h; 3 independent incubations per timepoint per compound), they were precipitated with 800 μL methanol. The samples were then gently shaken and put in the ultrasonic bath (0°C, 5 min). The mixture was then centrifuged at 4650 × *g* at 4°C for 5 min and 200 μL of the supernatant was removed for HPLC-MS/MS analysis. Quantitative analysis of fipronil and ivermectin was performed using liquid chromatography (Accela 600 pump, Accela AS autosampler) in combination with mass spectrometry LTQ-XL (all Thermo Fisher Scientific, San Jose, CA, USA). The chromatographic conditions were as follows: injection volume 5 μL; column Zorbax Eclipse Plus C18-Rapid Resolution HD (50 × 3 mm ID; 1.8 μm Agilent Technologies, Santa Clara, CA, USA) at 35°C; the mobile phase (A) 5 mM ammonium formate in methanol and (B) 5 mM ammonium formate in water; gradient change from A:B as follows: 0 min: 20:80, 4.5 min: 100:0, 8 min: 100:0, 8.1 min: 20:80, 10 min: 20:80 with a flow rate of 400 μL min^−1^. The conditions for mass spectrometry: negative (−2.5 kV) ion detection mode; capillary temperature 300°C, source heater temperature 300°C, sheath gas flow 35 au, aux gas flow 10 au, sweep gas flow 1 au. Eluted ions were detected in full scan mode from 200 to 1000 Da and in MS/MS mode. For the MS/MS analysis of ivermectin, the ion 919.6 Da [M + Formic-H]^-^ (NCE 24; 4 m/z), or 435.2 Da [M-H]^-^ (NCE 20; 4 m/z) for fipronil, was used (Fig. 1A). The data were acquired and processed using the XCalibur 4.0 software (Thermo Fisher Scientific).

### Preparation of *B. afzelii*-infected nymphs

Low-passage strain of *B. afzelii* (Pospisilova *et al*., 2019) was grown in Barbour-Stoenner-Kelly H (BSK-H) medium (Sigma-Aldrich, St. Louis, MO, USA) at 33°C for 5–7 days. Six-week-old female C3H/HeN mice were infected by subcutaneous injections of 10^5^ spirochetes per mouse. The presence of spirochetes in ear biopsies was verified 3 weeks post injection by PCR. Non-infected *I. ricinus* larvae were fed on *B. afzelii*-infected mice until repletion, allowed to moult to nymphs, and used for transmission experiments after 4–6 weeks.

### Mouse model transmission experiment with *B. afzelii*-infected *I. ricinus* nymphs

*Borrelia afzelii*-infected nymphs (~2 months after moulting) were placed on naive C3H/HeN mice (5 nymphs per mouse) and allowed to attach, which took ~2 h. Fipronil solution (15 mM; 0.7% w/v concentration) was then applied to the attached nymphs. In the control group, 20 μL of ethanol was applied to the attached nymphs. Each experimental group (fipronil and control group) contained 10 mice. Nymphs were allowed to feed; engorgement weights and feeding successes were recorded for each individual nymph. Four weeks after the challenge with tick nymphs, the mice were sacrificed by cervical dislocation, and ear, skin, bladder and heart biopsies were collected using sterile forceps and Metzenbaum scissors. The infection in murine tissues was determined by nested PCR amplification of a 222-bp fragment of a 23S rRNA gene as described previously (Pospisilova *et al*., 2019).

### Viability assay of *B. afzelii in vitro*, dark-field microscopy and immunodetection

Two hundred microliter of *B. afzelii* culture was distributed into sterile 1.5 mL tubes (33 tubes in total). Then fipronil or vancomycin (Merck, Sigma-Aldrich: SBR00001), a cell wall synthesis-blocking antibiotic (Wu *et al*., 2018), was added to the tubes at final concentrations of 500, 50, 5, 0.5, and 0.05 μg mL^−1^. Each concentration was tested in triplicate (30 tubes in total – 15 tubes for fipronil and 15 tubes for vancomycin). The cultures were then checked by immunofluorescence and dark-field microscopy on days 3, 5 and 7 after the addition of fipronil/vancomycin. For immunofluorescence detection, 50 μL of a *Borrelia* culture (7 days after addition of fipronil/vancomycin) was fixed with 4% paraformaldehyde on a SuperFrost® Plus (Thermo Scientific) slide for 20 min. The slides were then washed 3 times for 5 min each in phosphate-buffered saline (PBS) and permeabilized with 0.1% Triton X-100 (Tx) in PBS with 1% bovine serum albumin (Sigma) for 1 h at room temperature (RT). *Borrelia* were recognized with primary rabbit anti-*B. burgdorferi* antibody (1:200; Thermo Fisher Scientific, Invitrogen: PA1-73004) in PBS-Tx (0.1% Tx in PBS) for 90 min at RT. Slides were washed 2 × 10 min in PBS-Tx and stained with a secondary Alexa Fluor 488 goat anti-rabbit antibody (Thermo Fisher Scientific, Invitrogen: a11034), 1:500 in PBS-Tx, for 1 h at RT and washed 2 × 10 min in PBS. Slides were then mounted in DABCO and examined using an Olympus FluoView FV3000 confocal microscope (Olympus, Tokyo, Japan). Viability, motility and spirochete numbers were assessed and compared with control cultures (total of 3 tubes).

### Statistics and software

Data were analysed by GraphPad Prism 10, and an unpaired Student's *t*-test was used for evaluation of statistical significance. A *P* value of less than 0.05 was considered statistically significant. Error bars in the graphs show the standard errors of the means. Graphic arts were produced in BioRender.

## Results

### Fipronil and ivermectin are potent acaricides as assessed via oral administration

To evaluate the effects of commercial acaricides on tick feeding progression, fipronil and ivermectin were selected for this study. To qualify the selected compounds for the *ex vivo* artificial membrane-based blood feeding of ticks, we first evaluated the stability of fipronil and ivermectin in blood serum incubated at 37°C using LC-MS/MS (Fig. 1A). Both acaricides exhibited high thermal stability. Ivermectin retained full thermal resiliency, while fipronil showed a 40% thermal decay during 24 h incubation in blood serum (Fig. 1B). Both acaricides were added to the blood meal for the *ex vivo* feeding of ticks. The blood was replaced every 12 h to maintain consistent concentrations of acaricides in the blood meal. Both fipronil and ivermectin demonstrated highly potent acaricidal activity against *I. ricinus* females (Fig. 1C) and nymphs (Fig. 1D, Supplementary Figs S1 and S2). Even at nanomolar concentrations, these acaricides significantly impaired the ticks’ ability to fully engorge, highlighting their high efficacy at low doses. Given that fipronil gets rapidly metabolized to fipronil sulfone (Fig. 1E) in mice as well as other biological systems (Hainzl and Casida, 1996), which then lasts long in the body (Chang and Tsai, 2020), we also tested the toxicity of fipronil sulfone by supplementing it in the tick blood meal. Fipronil sulfone displayed a potent acaricidal activity against *I. ricinus* females (Fig. 1F, Supplementary Fig. S3 and Videos) and nymphs (Fig. 1G, Supplementary Fig. S3), inducing tick lethality at 250 nM and causing moribundity at 25 nM concentrations. Fipronil sulfone appears to be slightly, yet not significantly, more potent against *I. ricinus* females and nymphs at 25 nM when compared to pure fipronil (Fig. 1F and G).

### Fipronil has fast mode-of-action acaricidal activity upon topical administration

**Figure 2.**
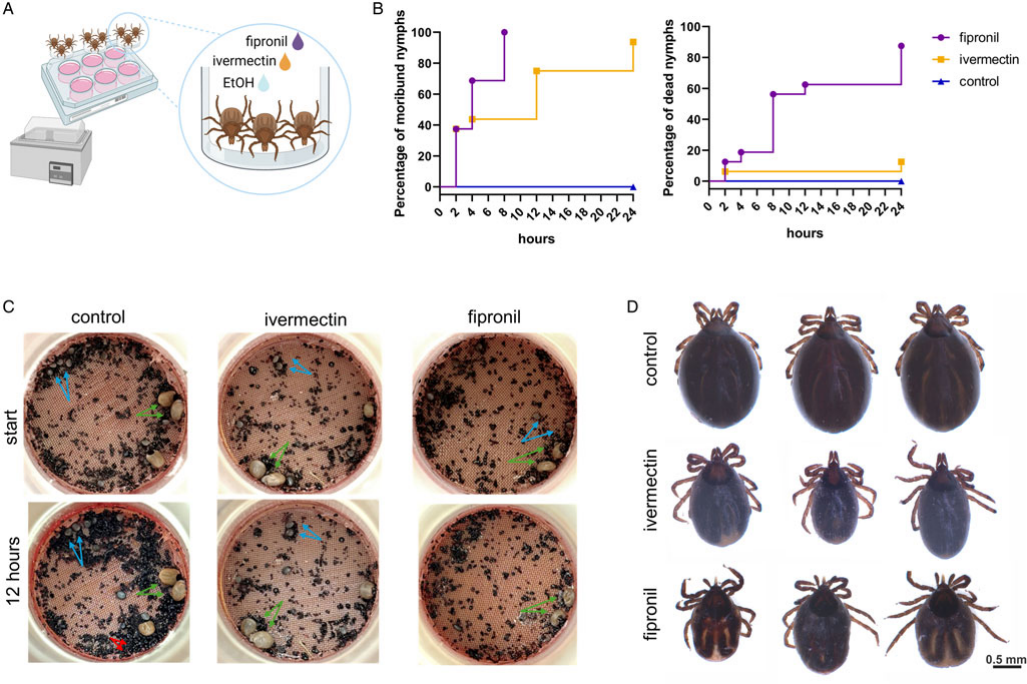
Temporal resolution of acaricidal impact on feeding ticks upon topical application. (A) A scheme of the *ex vivo* blood feeding experiment with *Ixodes ricinus* nymphs being exposed to topical treatment of fipronil, ivermectin, or ethanol (solvent control). (B) Impact curves of feeding *I. ricinus* nymphs in the *ex vivo* blood feeding system upon topical application of compounds. Ticks were monitored for their substandard moribund appearance (left) or their lethality (right) within the first 24 h upon exposure to the acaricide. Data were obtained from 2 independent blood feeding units per timepoint and compound. (C) Photographic images of representative feeding units before and 12 h after application of studied acaricidal compounds. Green arrows, *I. ricinus* females; blue arrows, *I. ricinus* nymphs; red arrows; ticks faeces (indicative of good blood feeding in control groups). (D) Photographic images of 3 representative individuals 24 h upon exposure to the acaricide.

To confirm the acaricidal activity upon topical application, *I. ricinus* nymphs were allowed to attach and feed on the silicone membrane in the *ex vivo* feeding system for 3 days before applying the acaricides (Fig. 2A). Shortly after application, both acaricides negatively impacted the status of attached nymphs. Nymphs treated with both acaricides displayed significant moribundity (mostly manifested by uncontrolled movements of legs) 12 h post-application (Fig. 2B, left; Fig. 2C). After 24 h, most fipronil-treated ticks were dead, while most ivermectin-treated ticks were moribund, and all control (ethanol-treated) ticks remained fully viable (Fig. 2B, right; Fig. 2D).

These data on topical toxicity demonstrate a substantially fast acaricidal power of both acaricides, especially fipronil, against *I. ricinus* nymphs. This raises the question of whether the fast-acting nature of these acaricides is sufficient to prevent the transmission of *B. afzelii* spirochetes, known to cause permanent infection in mice after ⩾24 h of tick feeding (Pospisilova *et al*., 2019).

### Topical application of fipronil prevents transmission of Lyme disease spirochetes in mice

**Figure 3.**
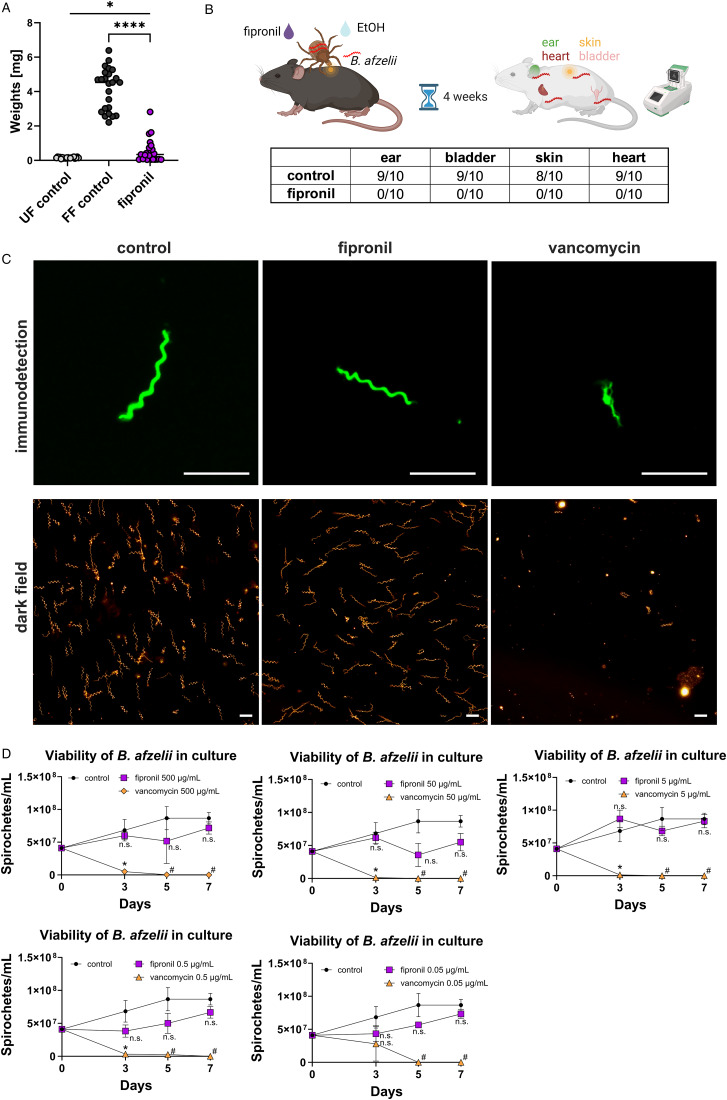
Fipronil prevents *Borrelia* transmission into mouse. (A) The graph shows the average weights of 20 individual *I. ricinus* nymphs. Weights were obtained on day 4 of feeding for fully-fed (FF) control and fipronil-treated nymphs; weights of unfed (UF) nymphs are also shown. Bars indicate standard errors of means. *T*-test *P* values: *=0.01, ****<0.0001. (B) PCR detection of spirochetes in mouse organs 4 weeks after exposure to 5 *B. afzelii*-infected *I. ricinus* nymphs treated with fipronil or solvent (ethanol) as a control. (C) Microscopic images of *Borrelia* cultured *in vitro* with fipronil or vancomycin (positive control) revealing the morphology of representative *B. afzelii* spirochetes (top panel, immunofluorescent detection) and their abundance (bottom panel, dark field). Scale bars represent 10 μm. (D) The graphs compare the survival of *B. afzelii* in culture treated with fipronil or vancomycin (500–0.05 μg mL^−1^) with an untreated control culture. Bars indicate standard errors of means; *t*-test: *P < 0.05, n.s. = not significant, # dead spirochetes.

The *in vivo* experiments explored the ability of fipronil to prevent *B. afzelii* transmission from infected *I. ricinus* nymphs to naive mice. Fipronil was applied topically on the ticks shortly after attachment to mice (application of fipronil before tick attachment leads to tick lethality before they can attach; data not shown) and ticks were allowed to fully engorge. The fipronil-treated ticks reached only a weight fraction of fully fed control ticks, yet significantly more than unfed ticks, indicating some extent of active blood feeding (Fig. 3A). Four weeks after tick feeding, biopsies of mouse ear, skin, heart and bladder were examined for the presence of *Borrelia*. Expectedly, 9 out of 10 mice challenged with solvent-treated control ticks were infected with *Borrelia*. Conversely, no *Borrelia* were detected in any organs of 10 mice exposed to fipronil-treated ticks (Fig. 3B). This 100% prevention of transmission in the fipronil group underscores the potential of rapid acaricidal effect of fipronil to eliminate ticks before successful *Borrelia* transmission can occur.

To rule out a direct effect of fipronil on *B. afzelii*, *in vitro* viability assays were conducted. *Borrelia afzelii* cultures supplemented with various concentrations of fipronil showed no effect on bacterial viability, unlike vancomycin, a positive control anti-borrelial antibiotic targeting bacterial cell wall synthesis (Wu *et al*., 2018) (Fig. 3C and D). These results demonstrate that fipronil does not directly affect *Borrelia* spirochetes and indicates that transmission is prevented through rapid and effective disruption of tick physiology.

## Discussion

Drug-based vector control holds great promise for reducing disease burden (Alout and Foy, 2016; Miglianico *et al*., 2018). Unlike blood-feeding insects, ticks (Ixodidae) exhibit prolonged association with their hosts, lasting several days. This extended feeding period creates a unique window of opportunity for intervention. The application of acaricides primarily targets the tick vector but also provides an opportunity to prevent pathogen transmission before it happens. The time required to transmit the minimum amount of pathogens capable of infecting the host is defined here as the minimum transmission time (MTT). The MTT values span across the scale from hours to days and are characteristic of individual tick species and pathogens they transmit, as illustrated by a concise overview of pathogens transmitted by *Ixodes* ticks (Table 1). The concept of exploiting acaricides as agents preventing pathogen transmission holds significant promise for reducing the risk of tick-borne diseases, such as Lyme disease caused by *Borrelia*.

**Table 1.** Overview of minimum transmission times (MTT)

Vector	Pathogen	Minimum transmission time	Reference*
*Ixodes ricinus*	*Borrelia afzelii*	⩾24 h	*a*, *b*
	*Borrelia burgdorferi*	>48 h	*b*
	*Borrelia miyamotoi*	Unknown	
	*Francisella tularensis*	Unknown	
	*Anaplasma phagocytophilum*	24–48 h	*c*, *d*
	*Babesia microti*	Unknown	
	TBE virus	>3 h	*e*
*Ixodes scapularis*	*Borrelia burgdorferi*	24–48 h	*f*
	*Borrelia miyamotoi*	<24 h	*g*
	*Francisella tularensis*	Unknown	
	*Anaplasma phagocytophilum*	<24 h	*h*
	*Babesia microti*	>36 h	*i*
	Powassan virus	15 min	*j*

Selected MTTs for tick–pathogen Pairs, as reported in the literature.

* References: *a* (Pospisilova *et al*., 2019), *b* (Crippa *et al*., 2002), c (Hodzic *et al*., 1998), *d* (Fourie *et al*., 2019), *e* (Thangamani *et al*., 2017), *f* (des Vignes *et al*., 2001), *g* (Breuner *et al*., 2017), *h* (Levin *et al*., 2021), *i* (Piesman and Spielman, 1980), *j* (Ebel and Kramer, 2004).

In earlier work, we established an initial framework for tick-targeted interventions to block *Borrelia* transmission with a series of RNAi experiments (Perner *et al*., 2022). While we were able to target essential transcripts by RNAi, which profoundly impeded tick blood-feeding success, the capacity of these RNAi-handicapped ticks to transmit *B. afzelii* spirochetes remained unchanged to controls (Perner *et al*., 2022). We hypothesize that the time needed for RNAi to become fully active exceeds the MTT value for the *I. ricinus*–*B. afzelii* couple. While RNAi is valuable for studying functions of tick proteins affecting the biology or life cycle of the pathogens (Hajdusek *et al*., 2023), it is less suitable for non-specific handicapping of ticks to study pathogen transmission.

In this work, we assessed the impact of commercially available chemical acaricides on tick biology and pathogen transmission capacity, as the interaction with tick molecules is immediate, modulating tick processes without delay. Both acaricides, fipronil or ivermectin, demonstrated high acaricidal potency against several tick species (Hunter *et al*., 2011; Dumont *et al*., 2015) and blood feeding poultry red mites (Ribeiro *et al*., 2023). While fipronil has clear activity against arthropods, *Borrelia* spirochetes were completely unaffected by fipronil supplementation *in vitro* (up to 1.14 mM fipronil). It indicates yet unknown resistance mechanism of *B. afzelii* to fipronil-mediated cytotoxicity, as fipronil reduces the viability of *Escherichia coli* at 100 μM concentration (Bhatti *et al*., 2019).

Fipronil is primarily formulated for external use, but its extraordinary environmental persistence (Bhatt *et al*., 2023) suggests a need for more directed administration. Subcutaneous or oral administration to heavily infested animals offers such an alternative (Cid *et al*., 2016; Poché *et al*., 2020, 2021, 2023*a*). While sulfoxide-containing phenylpyrazoles, such as fipronil, are short-lived in animal blood, their metabolic products, mainly fipronil sulfone, display a days-long half-life in vertebrates, retaining its insecticidal (Hainzl *et al*., 1998) and acaricidal activity, as demonstrated in this study. For acaricidal compounds to be successful candidates as effective transmission blockers, they must have a long half-life in biological systems and be available in vertebrate blood. Compounds from the isoxazoline class, exhibiting month-long half-lives (Toutain *et al*., 2017, 2018), are promising in this respect. Lotilaner (TP-05; Tarsus Pharmaceuticals, Inc.), an isoxazoline compound, has recently passed the Phase 2b trials, demonstrating its safety and concentration-dependent tick killing activity after they have attached to human skin (https://clinicaltrials.gov/). Data from this and other studies support the concept of acaricide-mediated protection of individuals from Borrelia transmission.

Other studies also assessed the impact of acaricides aimed at reducing pathogen transmission in smaller populations and restricted areas (Hinckley *et al*., 2016; Ostfeld *et al*., 2023). Under laboratory conditions, fipronil baits achieved 100% control of larval ticks on white-footed mice for up to 15 days post-treatment (Poché *et al*., 2020). Similarly, systemic acaricidal treatment with orally delivered fipronil significantly reduced the burden of juvenile *I. scapularis* on white-footed mice (Williams *et al*., 2023). Success in using ivermectin-treated corn to control ticks on an isolated deer population (Rand *et al*., 2000) or the high efficacy of fipronil formulations (Poché *et al*., 2023*b*, 2023*c*) in controlling ticks on white-tailed deer (*Odocoileus virginianus*) underscore the potential of systemic acaricides in integrated tick management programmes, particularly for reservoir animals. Conversely, it has been shown that the application of acaricides like bifenthrin (a pyrethroid) around residential properties decreased the number of questing ticks but did not reduce the number of tick exposures or the incidence of tick-borne diseases (Hinckley *et al*., 2016). This highlights the importance of targeting tick-pathogen transmission in individual end-hosts.

The practical implementation of tick population control in field settings requires further investigation and should be integrated with other tick management strategies. While acaricides show promise for reducing tick burdens on reservoir hosts, acaricide barriers have demonstrated limited efficacy in reducing household tick exposure and associated disease risk.

In conclusion, the extended feeding period of ticks compared to other blood-feeding insects creates a valuable window of opportunity for acaricidal intervention. This work establishes the proof-of-concept that a targeted approach offers a promising strategy for interrupting pathogen transmission and thus preventing tick-borne diseases such as Lyme borreliosis. We demonstrate the nanomolar efficiency of fipronil against *I. ricinus* ticks and its rapid speed-of-kill, setting a benchmark for the development of novel acaricides aimed at blocking the transmission of tick-borne pathogens.

## Supporting information

Šíma et al. supplementary material 1 Šíma et al. supplementary material

Šíma et al. supplementary material 2 Šíma et al. supplementary material

Šíma et al. supplementary material 3 Šíma et al. supplementary material

Šíma et al. supplementary material 4 Šíma et al. supplementary material

## Data Availability

All data are available in publication and supplementary material.

## Supplementary material

The supplementary material for this article can be found at https://doi.org/10.1017/S0031182024001136.
