# Supplementary material for: Fipronil prevents transmission of Lyme disease spirochetes
Source: Parasitology. 2024 Nov 20;151(9):953–61. doi: 10.1017/S0031182024001136 (PMC11770531; doi:10.1017/S0031182024001136)
Supplement: Šíma et al. supplementary material 1 — Šíma et al. supplementary material [file S0031182024001136sup001.docx]

**Fipronil prevents transmission of Lyme disease spirochetes**

Radek Šíma^1,2,*^, Adéla Palusová^1^, Tereza Hatalová^1^, Luise Robbertse^1^, Petra Berková^3^, Martin Moos^3^, Petr Kopáček^1^, Veronika Urbanová^1^, Jan Perner^1*^

^1^ *Institute of Parasitology, Biology Centre of the Czech Academy of Sciences, Branišovská 31, 370 05, České Budějovice, Czech Republic*

^2^ *Biopticka laborator, Mikulasske namesti 4, 32600 Plzen, Czech Republic*

^3^ *Institute of Entomology, Biology Centre of the Czech Academy of Sciences, Branišovská 31, 370 05, České Budějovice, Czech Republic*

* correspondence: sima@paru.cas.cz, perner@paru.cas.cz

**Supplementary Material**


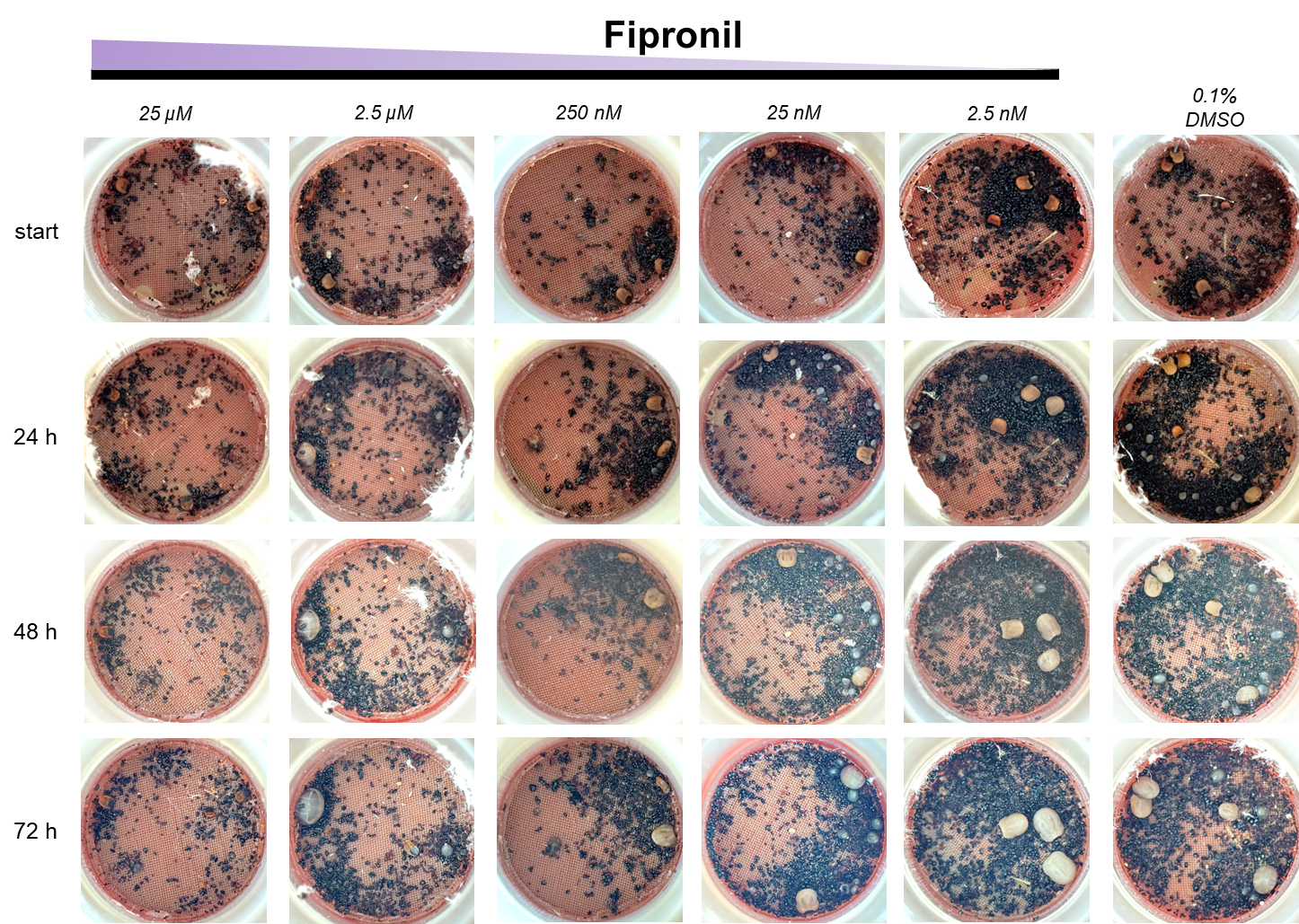


**Supplementary Figure S1.** Photographic images of the *ex vivo* membrane feeding system of ticks, monitoring blood feeding of *Ixodes ricinus* nymphs (together with pre-fed *I. ricinus* females to support nymphal feeding) in response to supplementation of blood meal with a concentration series of fipronil. Start denotes the start of a supplementation, i.e. Day 2 and Day 1 of feeding *I. ricinus* females and nymphs, respectively.


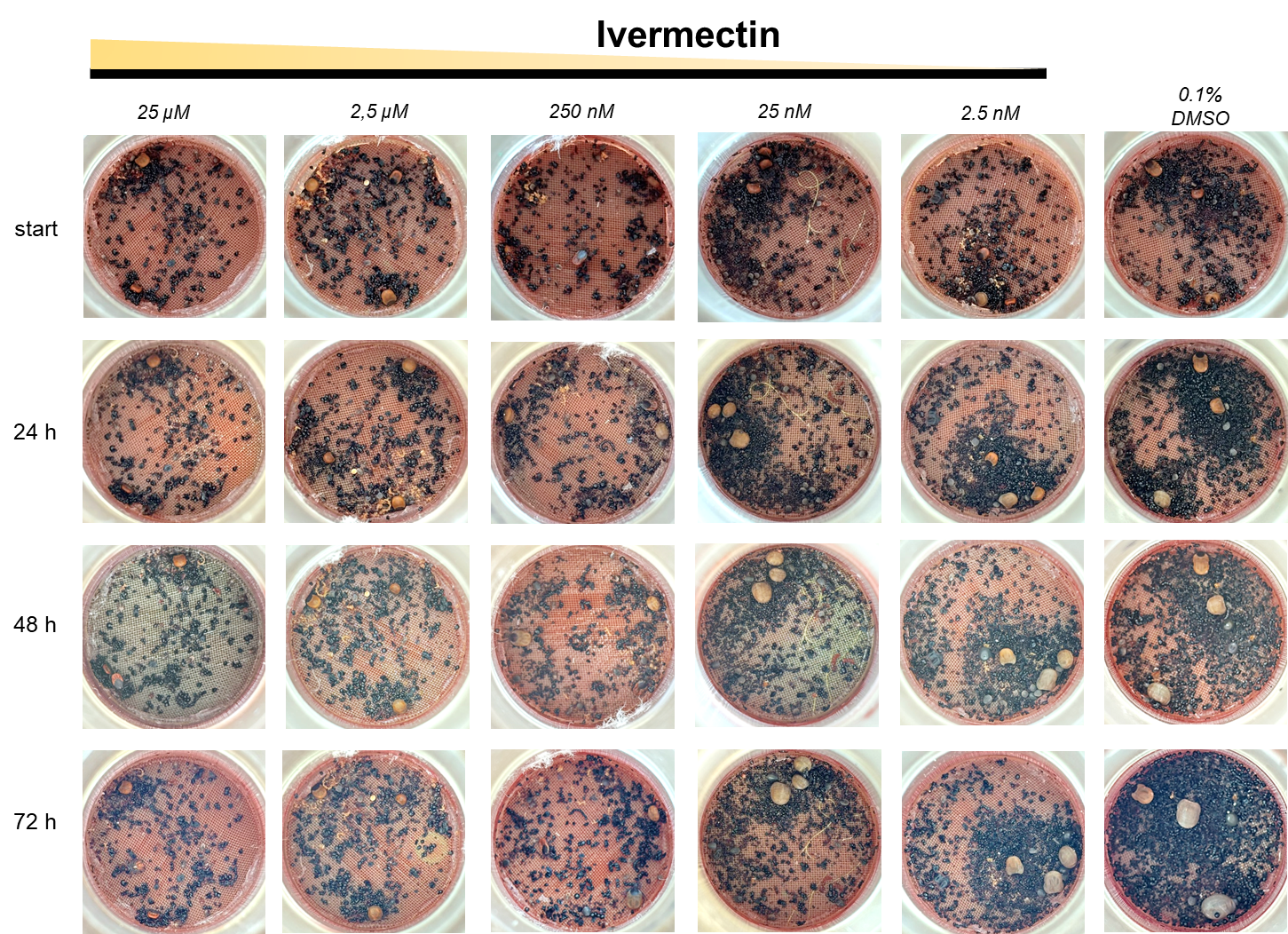


**Supplementary Figure S2.** Photographic images of the *ex vivo* membrane feeding system of ticks, monitoring blood feeding of *Ixodes ricinus* nymphs (together with pre-fed *I. ricinus* females to support nymphal feeding) in response to supplementation of blood meal with a concentration series of ivermectin. Start denotes the start of a supplementation, i.e. Day 2 and Day 1 of feeding *I. ricinus* females and nymphs, respectively.


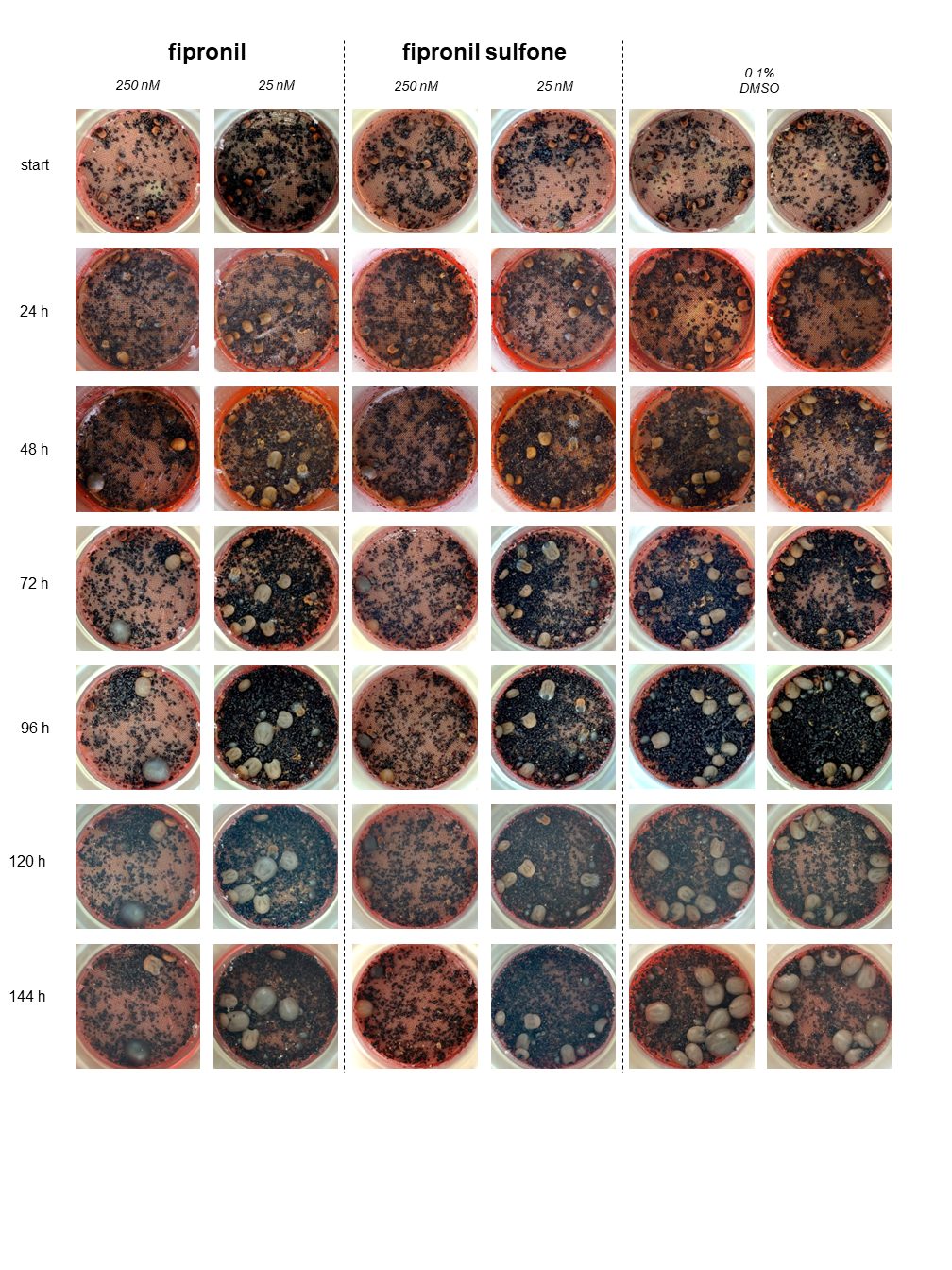


**Supplementary Figure S3.** Photographic images of the *ex vivo* membrane feeding system of ticks, monitoring blood feeding of *Ixodes ricinus* females and nymphs, combined in one feeding unit, in response to supplementation of blood meal with two concentrations (final) of fipronil and fipronil sulfone. Start denotes the start of a supplementation, i.e. Day 2 and Day 1 of feeding *I. ricinus* females and nymphs, respectively. Supplementary videos were taken from feeding units on Day 3 (72 hours) of supplementation.

**Supplementary Video 1.** A short video depicting an *ex vivo* membrane feeding system chamber containing *Ixodes ricinus* females and nymphs that have been feeding for five and four days, respectively. After 48 hours of feeding (of females), the blood meal was supplemented with 25 nM fipronil (final concentration) for the remaining duration of the feeding.

**Supplementary Video 2.** A short video depicting an *ex vivo* membrane feeding system chamber containing *Ixodes ricinus* females and nymphs that have been feeding for five and four days, respectively. After 48 hours of feeding (of females), the blood meal was supplemented with 25 nM fipronil sulfone (final concentration) for the remaining duration of the feeding.

**Supplementary Video 3.** A short video depicting an *ex vivo* membrane feeding system chamber containing *Ixodes ricinus* females and nymphs that have been feeding for five and four days, respectively. After 48 hours of feeding (of females), the blood meal was supplemented with 0.1% DMSO (final concentration) for the remaining duration of the feeding.
